# An association study between genetic polymorphisms related to lipoprotein-associated phospholipase A_2_ and coronary heart disease

**DOI:** 10.3892/etm.2013.911

**Published:** 2013-01-21

**Authors:** LIMIN XU, JIANQING ZHOU, STEPHANIE HUANG, YI HUANG, YANPING LE, DANJIE JIANG, FEIMING WANG, XI YANG, WEIFENG XU, XIAOYAN HUANG, CHANGZHENG DONG, LINA ZHANG, MENG YE, JIANGFANG LIAN, SHIWEI DUAN

**Affiliations:** 1 School of Medicine, The Affiliated Hospital, Ningbo University, Ningbo, Zhejiang 315211;; 2 Ningbo Medical Center, Lihuili Hospital, Ningbo, Zhejiang 315041, P.R. China;; 3 Department of Medicine, University of Chicago, Chicago, IL 60637, USA;; 4 Zhejiang Zhenhai Middle School, Ningbo, Zhejiang 315200, P.R. China

**Keywords:** coronary heart disease, lipoprotein-associated phospholipase A_2_, single nucleotide polymorphism, *CELSR2*, *PLA2G7*, *ZNF259*

## Abstract

Previous genome-wide association studies (GWAS) have revealed seven single nucleotide polymorphisms (SNPs) that affect lipoprotein-associated phospholipase A_2_ (Lp-PLA_2_) activity or levels in American and European individuals. A total of 290 coronary heart disease (CHD) patients, 198 non-CHD patients and 331 unrelated healthy volunteers were recruited for the present case-control study of Han Chinese. Four SNPs (rs964184 of *ZNF259*, rs7528419 of *CELSR2* and rs7756935 and rs1805017 of *PLA2G7*) were shown to be significantly associated with CHD. The rs964184-G allele of the *ZNF259* gene was identified as a risk factor of CHD in females (odds ratio (OR) =1.49, 95% confidence interval (CI) =1.00–2.22, P=0.05). The rs7528419-G allele of the *CELSR2* gene was protective against CHD in males (OR=0.48, 95% CI=0.25–0.93, P=0.04). The other two alleles (rs7756935-C and rs1805017-A) of the *PLA2G7* gene acted as protective factors against CHD in females (rs7756935-C: OR=0.59, 95% CI=0.35–1.00, P=0.05; rs1805017-A: OR=0.51, 95% CI=0.28–0.93, P=0.03). Moreover, rs1805017 of the *PLA2G7* gene was associated with the severity of CHD only in females (r^2^=0.02, P=0.04). We identified four Lp-PLA_2_-associated SNPs significantly associated with CHD in a Han Chinese population. Specifically, rs7528419 was protective factor against CHD in males, while the other two SNPs (rs7756935 and rs1805017 of the *PLA2G7* gene) were protective factors against CHD in females and rs964184 of the *ZNF259* gene was regarded as a risk factor for CHD in females.

## Introduction

Coronary heart disease (CHD) is a dynamic inflammatory disease process caused by atherosclerosis, which is the narrowing or blockage of the coronary arteries. Atherosclerosis may eventually lead to severe events, including sudden mortality, myocardial infarction (MI) or acute coronary syndrome (ACS). These events are often dependent on inflammatory changes within the arterial walls (1).

As a biomarker of plaque inflammation and stability (2), lipoprotein-associated phospholipase A_2_ (Lp-PLA_2_) plays an important role in the progression of atherosclerosis (3). Lp-PLA_2_ is a pro-inflammatory enzyme that hydrolyzes oxidized phospholipids to generate lysophosphatidylcholine and oxidized fatty acids (4). This enzyme non-covalently binds to plasma lipoproteins (5). Lp-PLA_2_ binds with a much higher affinity to low-density lipoprotein (LDL) than to high-density lipoprotein (HDL) (5). Approximately 80% of Lp-PLA_2_ is attached to LDL and the rest to HDL in plasma (2). LDL cholesterol (LDL-C) has a causal role in the development of cardiovascular disease (6). Epidemiological evidence has established a strong correlation between low levels of HDL cholesterol (HDL-C) and cardiovascular events (7). A 1 mg/dl increase in HDL-C results in a 3% reduction in CHD outcomes (8). CHD subjects often have abnormal phenotypes, including high LDL-C, low HDL-C and high triglyceride (TG) concentrations (9).

A meta-analysis identifed a high risk of cardiovascular events for patients with high Lp-PLA_2_ levels (2). Lp-PLA_2_ is important for the formation of an atherosclerotic plaque and its rupture (10). Another meta-analysis of ∼80,000 participants in 32 prospective studies identified that high levels of Lp-PLA_2_ mass and activity are associated with a risk of CHD, stroke and cardiovascular mortality (11). Lp-PLA_2_ activity has also been shown to be a predictive factor for stroke and transient ischemic attack (TIA) (12).

Grallert *et al*(13) identified seven Lp-PLA_2_-associated single nucleotide polymorphisms (SNPs), including rs7528419 of the *CELSR2* gene, rs7756935 and rs1805017 of the *PLA2G7* gene, rs964184 of the *ZNF259* gene, rs10846744 of the *SCARB1* gene, rs247616 of the cholesteryl ether transfer protein (*CETP)* gene and rs6511720 of the LDL receptor (*LDLR)* gene. Among them, rs1805017 and rs247616 are associated with Lp-PLA_2_ mass and the other five SNPs are associated with Lp-PLA_2_ activity. It has been suggested that these seven SNPs play an important role in either lipid levels or CHD. However there is a lack of evidence concerning their roles in CHD in a Han Chinese population. The aim of our study was to investigate the association between the seven Lp-PLA_2_-associated SNPs and CHD in Han Chinese individuals.

## Materials and methods

### Sample collection

A total of 488 unrelated individual inpatients were selected between May 2008 and November 2011 from Ningbo Lihuili Hospital, Zhejiang, China. Of these, 290 patients had CHD (males, 210; females, 80; age, 62.07±9.50 years) and 198 patients were non-CHD controls (males, 101; females, 97; age, 58.66±9.31 years). In addition, 331 healthy individuals from Ningbo, China were recruited as the healthy controls (males, 86; females, 245; age, 63.41±9.21 years). The patients had been examined by standardized coronary angiography according to the Seldinger method (14) and judged by at least two independent cardiologists. CHD cases (n=290) had ≥50% coronary artery occlusion of one or more of the major coronary arteries (15) or a history of prior angioplasty or coronary artery bypass surgery. Non-CHD controls (n=198) had <50% occlusion in the major coronary artery and did not have atherosclerotic vascular disease. All the samples were from Han Chinese individuals from Ningbo, China. All individuals had no cardiomyopathy or congenital heart, liver or renal disease. The blood samples of CHD cases, non-CHD controls and healthy controls were collected by the same investigators. Blood samples were collected in 3.2% citrate sodium-treated tubes and then stored at −80°C. The study protocol was approved by the Ethics Committee of Lihuili Hospital in Ningbo and informed written consent was obtained from all subjects.

### SNP genotyping

Human genomic DNA was prepared from peripheral blood samples using the nucleic acid extraction automatic analyzer (Lab-Aid 820, Xiamen, China) and was quantified using the PicoGreen^®^ dsDNA Quantification kit (Molecular Probes Inc., Eugene, OR, USA). Amplification was performed on the ABI GeneAmp^®^ PCR System 9700 Dual 384-Well Sample Block Module (Applied Biosystems, Foster City, CA, USA). Polymerase chain reaction (PCR) was performed with an initial denaturation stage at 94°C for 15 sec, followed by 45 cycles of 94°C for 20 sec, 56°C for 30 sec and 72°C for 1 min, then a final extension for 3 min at 72°C. Primer extension genotyping was performed on the Sequenom MassARRAY iPLEX^®^ platform (Sequenom Inc., San Diego, CA, USA) according to the manufacturer’s instructions (16). The primer extension reaction included an initial denaturation stage at 94°C for 30 sec, followed by 40 cycles of amplification, including 94°C for 5 sec, 5 cycles at 52°C for 5 sec and 80°C for 5 sec, then a final extension for 3 min at 72°C. After purifying the products and transferring to a SpectroCHIP, MALDI-time-of-flight (TOF) mass spectrometry was used for SNP genotyping.

### Statistical analysis

The Hardy-Weinberg equilibrium (HWE) was analyzed using Arlequin software (v3.5) (17). Genotype and allele frequencies between CHD cases and each of the two controls (non-CHD controls and healthy controls) were compared using Clump 16 software with 10,000 Monte Carlo simulations (18). The linkage disequilibrium (LD) between the two SNPs in *PLA2G7* was measured by an online calculator (http://www.oege.org/software/cubex/). The odds ratio (OR) with 95% confidence interval (95% CI) were calculated using an online program (http://faculty.vassar.edu/lowry/odds2x2.html). The power of the study was estimated by the Power and Sample Size Calculation software (v3.0.43) (19). The logistic regression test between genotype and the severity of CHD was performed using R statistical software. A two-tailed P-value <0.05 was considered to indicate a statistically significant difference.

## Results

### HWE test

In the present study, we examined a total of seven SNPs associated with either the mass or the activity of Lp-PLA_2_. We first analyzed the HWE of these SNPs in CHD cases, non-CHD controls and healthy controls. All SNPs were consistent with HWE with the exception of two SNPs (rs10846744 and rs1805017). The rs10846744 SNP of the *SCARB1* gene did not meet HWE in the CHD cases and in each of the two controls (P<0.001) and thus was not further analyzed. The genotype distribution of rs1805017 of the *PLA2G7* gene did not meet the HWE in the non-CHD controls (P=0.01). There was a departure from HWE for rs1805017 in non-CHD male controls (P=0.01); however, its genotype frequencies in non-CHD female controls met HWE (P=0.23). Therefore, the association between rs1805017 and Lp-PLA_2_ was only studied in females.

### Genotypic and allelic analyses

The genotype and allele frequencies of six SNPs are shown in Table I. Although no significant difference was observed in the genotype level for all the SNPs, there was a significant difference in allele frequency distribution of two SNPs (rs7756935 and rs964184). The allele frequency of rs7756935-C was significantly higher in healthy controls than in CHD cases (17.2 vs. 12.9%; P= 0.04, OR= 0.72, 95% CI= 0.52-0.98). A significant association between rs964184 and CHD was observed in CHD cases compared with non-CHD controls (P=0.04, OR=1.40, 95% CI=1.03–1.90). The HWE test of rs964184 in healthy controls yielded a P-value of 0.06; therefore, it is possible that an association exists between rs964184 and CHD, compared with healthy controls.

### LD and haplotype test

We performed an LD test of rs7756935 and rs1805017 of the *PLA2G7* gene in CHD cases, non-CHD controls and healthy controls. Our results indicated that the two SNPs were in high, but not complete LD (D’=1.0, r^2^<0.05). Since there was a departure from HWE for rs1805017 in males, we only performed an LD test and haplotype test for the two SNPs in females. As shown in Table II, the haplotype rs7756935A-rs1805017G significantly increased the risk of CHD in females (CHD cases vs. non-CHD controls: P=0.04, OR=1.64, 95% CI=1.02–2.65; CHD cases vs. healthy controls: P=0.003, OR=1.88, 95% CI=1.24–2.85).

### Genetic testing under the dominant and recessive inheritance models

Single SNP analyses for all six SNPs in HWE were performed under the dominant and recessive inheritance models. In the dominant model (Table III), significant associations between rs7756935 and rs964184 with CHD were observed between CHD cases and healthy controls. The rs7756935-C allele was a protective factor for CHD (P=0.03, OR=0.68, 95% CI=0.48–0.97) and rs964184-G was a risk factor for CHD (P=0.04, OR= 1.40, 95% CI=1.01–1.93). A trend of association between rs964184 and CHD was observed between CHD cases and non-CHD controls (P=0.06, OR=1.43, 95% CI=0.99–1.53). The six SNPs were not significantly associated with CHD in the recessive model (data not shown).

### Genetic testing stratified by gender

CHD is the leading cause of mortality worldwide for males and females. However, higher incidences have been observed in males compared with females at all ages and coronary disease occurs up to 10 years later in females (20). Gender issues have received increasing attention in international health policy. Due to the genetic and habitual differences between males and females, we further examined the roles of these SNPs in males and females separately.

In the present study, we performed a gender-stratified analysis to investigate whether gender influences the contribution of SNPs to the risk of CHD. The genotypic and allelic levels are shown in Table IV and V for males and females, respectively.

In the male-stratified samples, rs7528419 of the *CELSR2* gene presented a significant association with CHD in CHD cases compared with healthy controls (genotypic, P=0.03; allelic, P=0.04). However, these significant results were not replicated in females (genotypic, P=0.56; allelic, P=0.56), suggesting a gender-dependent effect of rs7528419. In females, rs7756935 of the *PLA2G7* gene had a significant association with CHD in CHD cases compared with healthy controls (allelic, P=0.05, OR=0.59, 95% CI=0.35–1.00). The rs1805017 SNP of *PLA2G7* had a significant association with CHD in CHD cases compared with non-CHD controls (P=0.03, OR=0.51, 95% CI=0.28–0.93). A tendency of association was observed for rs964184-G of the *ZNF259* gene between CHD cases and non-CHD controls (allelic P=0.06, OR=1.60, 95% CI=0.99–2.58). This result was also observed between female CHD cases and female healthy controls (allelic, P=0.05, OR=1.49, 95% CI=1.00–2.22).

### Gender-stratified analyses under the dominant and recessive inheritance models

We explored the dominant and recessive inheritance models in male- and female-stratified analyses, respectively. Our results between CHD cases and healthy controls revealed that rs7528419-G is a protective factor for CHD in males (P=0.05, OR=0.49, 95% CI=0.24–0.98). No other significant results were observed in males for the other SNPs (data not shown).

In females (Table VI), rs7756935-C of the *PLA2G7* gene had a tendency as a protective factor for CHD in the association test comparing CHD cases and healthy controls (P=0.07, OR=0.59, 95% CI=0.33–1.07). The rs964184-G allele of the *ZNF259* gene had a tendency as a risk factor for CHD in the association test comparing CHD cases and non-CHD controls (P=0.07, OR=1.79, 95% CI=0.98–3.27). This finding was also observed when comparing CHD cases and healthy controls (P=0.03, OR=1.75, 95% CI=1.05–2.90).

### Correlation between genotypes and the severity of CHD

According to the angiographic evidence, CHD cases with ≥50% coronary artery occlusion in one, two and three or more coronary arteries were divided into three respective subgroups. Logistic regression analysis was performed using R statistical software. The P-values indicated the correlation between the severity of CHD and SNPs (Table VII). The rs1805017 SNP of the *PLA2G7* gene was significantly associated with the severity of CHD in females (P=0.04). In addition, rs964184 of the *ZNF259* gene had a marginal association with the severity of CHD (P=0.05).

## Discussion

As an inflammatory enzyme expressed in atherosclerotic plaques, Lp-PLA_2_ has become a therapeutic target in trials of vascular disease prevention (2). A prospective epidemiological study investigated the associations between circulating Lp-PLA_2_ and the risk of vascular diseases (11). Evidence has shown that Lp-PLA_2_ is involved in the pathogenesis of atherosclerotic plaque progression (10). It has been regarded as a inflammatory biomarker to predict the risk of stroke and MI (21).

Genetic variations of the Lp-PLA_2_ gene reduces or eliminates enzyme activity (5). Twenty-five epidemiological studies have established that Lp-PLA_2_ is a unique vascular-specific biomarker of plaque instability and rupture (10). A meta-analysis of 20,000 patients indicated a high risk for cardiovascular events in patients with high Lp-PLA_2_ levels (2). In light of the above evidence, we performed a case-control study to investigate the contribution of seven SNPs related to Lp-PLA_2_, to the risk of CHD.

The rs7528419 SNP of the *CELSR2* gene influences the risk of CHD by regulating the level of plasma LDL-C (22). One study demonstrated that *CELSR2* gene variants are strongly associated with LDL-C and Lp-PLA_2_ activity (5). The rs599839 SNP in the vicinity of the *PSRC1* and *CELSR2* genes may enhance CHD risk by regulating plasma LDL-C level (22). Zhou *et al* explored the association of five lipid-associated SNPs with CHD in Chinese individuals (23). The rs599839 SNP in *CELSR2*-*PSRC1*-*SORT* is a novel SNP associated with reduced CHD risk in Chinese individuals (OR= 0.076, 95% CI=0.61–0.90, P=0.001, in the dominant model) (23). In the present study, we identified rs7528419-G as a protective factor for CHD in the male-stratified association test between the CHD cases and the healthy controls (OR=0.48, 95% CI=0.25–0.93, P=0.04). The association is not supported in females, suggesting an effect of gender in rs7528419. Since the size of the male samples was not large (male CHD cases, n=210; male non-CHD control, n=101; male healthy controls, n=86) in our study, further replication of rs7528419 is warranted.

As the encoding gene of the Lp-PLA_2_ enzyme, *PLA2G7* has been frequently studied and controversy remains in the genetic association between this gene and CHD. A meta-analysis of 12 studies (10,494 cases and 15,624 controls) presented a negative association of *PLA2G7* variants with cardiovascular risk factors, coronary atheroma or CHD (24), while several other studies have demonstrated a correlation between *PLA2G7* and CHD (25–27). A Genecard Study identified that *PLA2G7* represents an important and potentially functional factor in the pathophysiology of CHD (25). Among the 19 SNPs studied, R92H (rs1805017) and A379V (rs1051931) have become the two most significant SNPs associated with CHD (25). In Chinese individuals, another two *PLA2G7* SNPs have been shown to be significantly associated with CHD, including V279F (rs16874954) (26). A379V (rs1051931) has been shown to have a negative association with the risk of CHD in Chinese individuals (26). In a South Korean population, the presence of the 279F allele reduces the risk of CVD (OR=0.646, 95% CI=0.490–0.850, P=0.002). No significant association has been identified between the A379V genotype and CVD risk in South Korean individuals (27). Carriage of the *PLA2G7* 279F allele causes a natural deficiency in Lp-PLA_2_ activity and thus has been treated as a protective factor against CHD in Korean males (OR=0.80, 95% CI=0.66–0.97, P=0.02) (28). Since V279F and A379V have been investigated in Chinese individuals (26), the other two SNPs (rs7756935 and rs1805017) of the *PLA2G7* gene were investigated in the present study.

The two SNPs (rs7756935 and rs1805017) of the *PLA2G7* gene are associated with Lp-PLA_2_ activity or mass (13); however, there is no direct link between them and CHD. Our association study between the CHD cases and the healthy controls identified that rs7756935 of the *PLA2G7* gene is associated with the risk of CHD on the allelic level (OR=0.72, 95% CI=0.52–0.98, P=0.04) and in the dominant model (CA+CC vs. AA, OR=0.68, 95% CI=0.48–0.97, P=0.03). There was no distribution difference among the male subgroups; however, a borderline significant association on the allelic level was observed between the CHD cases and the healthy controls in the female-stratified test (OR=0.59, 95% CI=0.35–1.00, P=0.05). The rs7756935-C allele is regarded as a protective factor for CHD in females, although this finding requires further confirmation.

For the rs1805017 SNP of the *PLA2G7* gene, HWE was met in female non-CHD controls; however, this was not the case in male non-CHD controls. The rs1805017-A allele had a significantly different distribution between CHD cases and non-CHD controls in the female-stratified association test (OR=0.51, 95% CI=0.28–0.93, P=0.03). This result is consistent with the findings by Sutton *et al* (OR=0.69, 95% CI=0.56–0.85, P<0.001) (25). Logistic regression analysis indicated that rs1805017 may be related to the progressive stages of CHD. In addition, we observed that the haplotype rs7756935A–rs1805017G significantly increases the risk of CHD in females (CHD cases vs. non-CHD controls: P=0.04, OR=1.64, 95% CI=1.02–2.65; CHD cases vs. healthy controls: P=0.003, OR=1.88, 95% CI=1.24–2.85).

The rs964184 SNP of the *ZNF259* gene region is near the gene cluster *APOA5-APOA4-APOC3-APOA1*. The ZNF259 protein strongly increases Lp-PLA_2_ activity and TG level (29). The rs964184 SNP is associated with HDL-C in subjects with atherogenic dyslipidemia (30). Atherogenic dyslipidemia is a syndrome with a high risk of CHD, characterized by elevated TG, decreased HDL-C and elevated LDL-C (31). A meta-analysis of 8 studies demonstrated that rs964184-G significantly increases the risk of CHD (OR=1.22, 95% CI=1.14–1.30, P=1.2×10^−9^) (32). Consistent with previous findings, our results demonstrated that the rs964184-G allele exerts a risk of CHD in Han Chinese individuals. In addition, logistic regression analysis for rs964184 revealed an association with CHD severity (P=0.05).

The *SCARB1* gene is a key lipoprotein receptor involved in the reverse cholesterol transport pathway (5). Previous studies demonstrated that the *SCARB1* gene is associated with HDL-C levels and thus regulates inflammatory responses (33). A plausible genetic interaction was identified between *SCARB1* gene polymorphisms and the risk of CHD in male individuals (34). Another study indicated that *SCARB1* null female mice experience accelerated atherosclerosis (33). The rs10846744 SNP of the *SCARB1* gene is associated with HDL-C levels (5) and the risk of CHD (13). The rs10846744 SNP of the *SCARB1* gene is also associated with atherosclerosis in multiple ethnic populations, including African American (P=0.03), Chinese (P=0.02), European American (P=0.05) and Hispanic populations (P=0.03) (30). The rs10846744-C allele has an association with higher common carotid intimal-medial artery thickness (CCIMT) in Chinese individuals (P=0.02) (30). Our study identified a strong departure from HWE for rs10846744 of the *SCARB1* gene (P<0.001). The possible reasons for this phenomenon in our study may be the improperly pair-wise design or the small population size. The subjects in this study originated from Ningbo, China and none of them were related. This SNP is not further analyzed in this study due to the departure from HWE.

The rs247616 SNP is located on the promoter region of the *CETP* gene (35), which is associated with the activity of CETP in regulating the metabolism of HDL-C (36). The low concentrations of HDL-C have been regarded as an independent risk factor for cardiovascular disease (9). Increasing the level of HDL-C is under consideration as a secondary therapeutic target for CHD patients (9). In the Whitehall II study, rs247616 was shown to be independently associated with increased HDL-C levels in males (P=9.6E-28) (35). A genome-wide association study (GWAS) revealed that rs247616 of the *CETP* gene is a quantitative trait locus of HDL level (P=9.7×10^−24^) (37). *CETP* gene variants may affect coronary risk via mechanisms unrelated to HDL-C level (38). In the present study, we were unable to observe a significant association between rs247616 of the *CETP* gene and CHD (P>0.05). This may be explained by an ethnic difference in the Chinese population or a lack of power in our samples.

The rs6511720 SNP is located on intron 1 of the *LDLR* gene and is associated with a lower risk of MI (39). *LDLR* mutations often cause autosomal dominant hypercholesterolemia (ADH) by affecting the hepatic clearance of blood LDL-C (40), Notably, ADH is clinically characterized by high blood LDL-C and atherosclerosis that may eventually lead to CHD (41). The majority of Lp-PLA_2_ is attached to LDL; however, the known genetic determinants of LDL-C levels, including the *LDLR* locus, are not significantly associated with CHD. ADH is commonly caused by mutations in the *LDLR*, apolipoprotein B-100 (*APOB*) or proprotein cinvertase subtilisin kexine 9 (*PCSK9*) genes (42). ADH is characterized by a high concentration of plasma LDL-C and increased risk of premature CHD (40). The association of *LDLR* and ADH is well studied. We are unable to replicate the correlation between rs6511720 of the *LDLR* gene and CHD (P>0.05). Further analysis of other *LDLR* variants is required.

Although a total of 819 Han Chinese individuals were included in our study, it is still not well powered, since the strongest power is 64.3% for rs7756935. The structure of gender in our samples should be adjusted in the future to ensure a more balanced case-control study. All the P-values provided in this study are not corrected by the number of tests, thus there is a chance of false positive results for our study.

In summary, we identified significant associations between four SNPs (rs7528419 of *CELSR2*, rs7756935 and rs1805017 of *PLA2G7* and rs964184 of *ZNF259*) and CHD in Han Chinese individuals. The rs7528419-G allele reduces the risk of CHD in males. Two SNPs (rs7756935 and rs1805017) in the *PLA2G7* gene act as protective factors in females, while rs964184-G is regarded as a risk factor for CHD.

## Figures and Tables

**Table I.** Genotype and allele analysis of the six SNPs.

SNPs	n	Genotype(n)			χ^2^	P-value	HWE	Allele (n)		χ^2^	P-value	OR (95% CI)
rs7528419 (*CELSR2*)		AA	AG	GG				A	G			
CHD cases	290	258	32	0			1.00	548	32			
Non-CHD controls	198	178	19	1	1.71	0.45	0.43	375	21	0.02	0.89	1.04 (0.59–1.84)
Healthy controls	331	288	42	1	1.30	0.58	1.00	618	44	0.69	0.47	0.82 (0.51–1.31)
rs7756935 (*PLA2G7*)		AA	CA	CC				A	C			
CHD cases	289	219	65	5			1.00	503	75			
Non-CHD controls	198	147	49	2	0.72	0.73	0.54	343	53	0.03	0.92	0.97 (0.66–1.41)
Healthy controls	331	225	98	8	4.63	0.10	0.57	548	114	4.30	0.04	0.72 (0.52–0.98)
rs1805017 (*PLA2G7*)		GG	GA	AA				G	A			
CHD cases	288	198	83	7			0.83	479	97			
Non-CHD controls	197	135	49	13	5.60	0.07	0.01	319	75	0.77	0.40	0.86 (0.62–1.20)
Healthy controls	331	220	103	8	0.39	0.81	0.45	543	119	0.28	0.66	0.92 (0.69–1.24)
rs964184 (*ZNF259*)		CC	GC	GG				C	G			
CHD cases	290	156	114	20			1.00	426	154			
Non-CHD controls	197	123	67	7	4.78	0.09	0.67	313	81	4.60	0.04	1.40 (1.03–1.90)
Healthy controls	331	205	103	23	4.73	0.10	0.06	513	149	2.74	0.10	1.25 (0.96–1.61)
rs247616 (*CETP*)		CC	TC	TT				C	T			
CHD cases	289	204	74	11			0.20	482	96			
Non-CHD controls	198	133	58	7	0.81	0.66	0.81	324	72	0.41	0.55	0.90 (0.64–1.26)
Healthy controls	330	249	71	10	1.87	0.40	0.10	569	91	1.91	0.19	1.25 (0.91–1.70)
rs6511720 (*LDLR*)		GG	GT	TT				G	T			
CHD cases	289	282	7	0			1.00	571	7			
Non-CHD controls	198	196	2	0	1.29	0.31	1.00	394	2	1.28	0.33	2.42 (0.50–11.69)
Healthy controls	331	323	8	0	0.00	1.00	1.00	654	8	0.00	1.00	1.00 (0.36–2.78)

SNPs, single nucleotide polymorphisms; CHD, coronary heart disease; HWE, Hardy-Weinberg equilibrium; OR, odds ratio; CI, confidence interval; CETP, cholesteryl ether transfer protein; LDLR, low-density lipoprotein receptor.

**Table II.** Estimated haplotypes in female cases and controls.

rs7756935	rs1805017	CHD cases (n=160)	Non-CHD controls (n=192)	OR (95% CI)	P-value	Healthy controls (n=490)	OR (95% CI) P-value	
A	A	17	40	0.45 (0.25–0.83)	0.01	82	0.59 (0.34–1.03)	0.080
A	G	124	130	1.64 (1.02–2.65)	0.04	317	1.88 (1.24–2.85)	0.003
C	G	17	22	0.92 (0.47–1.80)	0.87	91	0.52 (0.30–0.91)	0.020
C	A	2	0	-	-	0	-	-

CHD, coronary heart disease; OR, odds ratio; CI, confidence interval.

**Table III.** Differences in the genotype distributions under the dominant model.

	CHD cases vs. non-CHD controls		CHD cases vs. healthy controls	
Dominant model	OR (95% CI)	P-value	OR (95% CI)	P-value
rs7528419 (AG+GG vs. AA)	1.10 (0.61–1.99)	0.76	0.85 (0.52–1.39)	0.54
rs7756935 (CA+CC vs. AA)	0.92 (0.61–1.40)	0.75	0.68 (0.48–0.97)	0.03
rs1805017 (GA+AA vs. GG)	0.99 (0.67–1.30)	1.00	0.91 (0.65–1.28)	0.55
rs964184 (GC+GG vs. CC)	1.43 (0.99–1.53)	0.06	1.40 (1.01–1.93)	0.04
rs247616 (TC+TT vs. CC)	0.90 (0.69–1.17)	0.43	1.28 (0.90–1.83)	0.20
rs6511720 (GT+TT vs. GG)	2.43 (0.50–11.83)	0.31	1.00 (0.36–2.79)	1.00

CHD, coronary heart disease; OR, odds ratio; CI, confidence interval.

**Table IV.** Frequencies of the genotypes and alleles for the various SNPs in males.

SNPs	n	Genotype (n)			χ^2^	P-value	HWE	Allele (n)		χ^2^	P-value	OR (95% CI)
rs7528419 (*CELSR2*)		AA	AG	GG				A	G			
CHD cases	210	189	21	0			1.00	399	21			
Non-CHD controls	101	94	6	1	3.44	0.16	0.13	194	8	0.33	0.69	1.28 (0.56–2.93)
Healthy controls	86	70	15	1	5.74	0.03	0.58	155	17	4.85	0.04	0.48 (0.25–0.93)
rs7756935 (*PLA2G7*)		AA	CA	CC				A	C			
CHD cases	209	158	46	5			0.38	362	56			
Non-CHD controls	101	72	27	2	0.87	0.67	1.00	171	31	0.43	0.55	0.85 (0.53–1.37)
Healthy controls	86	65	19	2	0.00	1.00	0.64	149	23	0.00	1.00	1.00 (0.59–1.69)
rs1805017 (*PLA2G7*)		GG	GA	AA				G	A			
CHD cases	208	136	66	6			0.65	338	78			
Non-CHD controls	101	73	21	7	6.01	0.04	0.01	167	35	0.18	0.74	1.10 (0.71–1.71)
Healthy controls	86	51	33	2	1.22	0.58	0.34	135	37	0.59	0.49	0.84 (0.54–1.31)
rs964184 (*ZNF259*)		CC	GC	GG				C	G			
CHD cases	210	118	79	13			1.00	315	105			
Non-CHD controls	100	63	35	2	3.12	0.22	0.35	161	39	2.30	0.15	1.38 (0.91–2.08)
Healthy controls	86	55	25	6	1.96	0.37	0.20	135	37	0.81	0.40	1.22 (0.79–1.86)
rs247616 (*CETP*)		CC	TC	TT				C	T			
CHD cases	209	148	53	8			0.31	349	69			
Non-CHD controls	101	66	33	2	2.33	0.33	0.51	165	37	0.31	0.66	0.88 (0.57–1.06)
Healthy controls	86	68	16	2	2.16	0.38	0.31	152	20	2.26	0.17	1.50 (0.88–2.56)
rs6511720 (*LDLR*)		GG	GT	TT				G	T			
CHD cases	209	205	4	0			1.00	414	4			
Non-CHD controls	101	101	0	0	1.96	0.31	1.00	202	0	1.95	0.31	-
Healthy controls	86	83	3	0	0.65	0.68	1.00	169	3	0.64	0.68	0.54 (0.12–2.46)

SNPs, single nucleotide polymorphisms; HWE, Hardy-Weinberg equilibrium; OR, odds ratio; CI, confidence interval; CHD, coronary heart disease; CETP, cholesteryl ether transfer protein; LDLR, low-density lipoprotein receptor.

**Table V.** Frequencies of the genotypes and alleles for the various SNPs in females.

SNPs	n	Genotype (n)			χ^2^	P-value	HWE	Allele (n)		χ^2^	P-value	OR (95% CI)
rs7528419 (*CELSR2*)		AA	AG	GG				A	G			
CHD cases	80	69	11	0			1.00	149	11			
Non-CHD controls	97	84	13	0	0.01	1.00	1.00	181	13	0.00	1.00	1.03 (0.45–2.36)
Healthy controls	245	218	27	0	0.44	0.56	1.00	463	27	0.41	0.56	1.27 (0.61–2.61)
rs7756935 (*PLA2G7*)		AA	CA	CC				A	C			
CHD cases	80	61	19	0			0.59	141	19			
Non-CHD controls	98	75	22	0	0.03	1.00	0.60	172	22	0.02	1.00	1.05 (0.55–2.02)
Healthy controls	245	160	79	6	4.46	0.10	0.40	399	91	3.85	0.05	0.59 (0.35–1.00)
rs1805017 (*PLA2G7*)		GG	GA	AA				G	A			
CHD cases	80	62	17	1			1.00	141	19			
Non-CHD controls	96	62	28	6	4.85	0.10	0.23	152	40	5.02	0.03	0.51 (0.28–0.93)
Healthy controls	245	169	70	6	2.23	0.37	0.82	408	82	2.17	0.17	0.67 (0.39–1.14)
rs964184 (*ZNF259*)		CC	GC	GG				C	G			
CHD cases	80	38	35	7			1.00	111	49			
Non-CHD controls	97	60	32	5	3.81	0.17	0.77	152	42	3.70	0.06	1.60 (0.99–2.58)
Healthy controls	245	150	78	17	4.69	0.09	0.15	378	112	3.91	0.05	1.49 (1.00–2.22)
rs247616 (*CETP*)		CC	TC	TT				C	T			
CHD cases	80	56	21	3			0.69	133	27			
Non-CHD controls	97	67	25	5	0.20	0.93	0.19	159	35	0.08	0.78	0.92 (0.53–1.60)
Healthy controls	244	181	55	8	0.54	0.79	0.19	417	71	0.51	0.52	1.19 (0.73–1.94)
rs6511720 (*LDLR*)		GG	GT	TT				G	T			
CHD cases	80	77	3	0			1.00	157	3			
Non-CHD controls	97	95	2	0	0.46	0.66	1.00	192	2	0.45	0.67	1.83 (0.30–11.12)
Healthy controls	245	240	5	0	0.73	0.41	1.00	485	5	0.72	0.41	1.85 (0.44–7.84)

SNPs, single nucleotide polymorphisms; HWE, Hardy-Weinberg equilibrium; OR, odds ratio; CI, confidence interval; CHD, coronary heart disease; CETP, cholesteryl ether transfer protein; LDLR, low-density lipoprotein receptor.

**Table VI.** Differences in genotype distributions under the dominant and recessive models in females.

A, Dominant model.				
	CHD cases vs. non-CHD controls		CHD cases vs. healthy controls	
	OR (95% CI)	P-value	OR (95% CI)	P-value
rs7528419 (AG+GG vs. AA)	1.03 (0.43–2.44)	1.00	1.29 (0.61–2.73)	0.55
rs7756935 (CA+CC vs. AA)	1.06 (0.53–2.14)	1.00	0.59 (0.33–1.05)	0.07
rs1805017 (GG vs. GA+AA)	0.53 (0.27–1.04)	0.07	0.65 (0.36–1.17)	0.16
rs964184 (GC+GG vs. CC)	1.79 (0.98–3.27)	0.07	1.75 (1.05–2.90)	0.03
rs247616 (TC+TT vs. CC)	0.96 (0.50–1.82)	1.00	1.23 (0.71–2.15)	0.48
rs6511720 (GT+TT vs. GG)	1.85 (0.30–11.36)	0.66	1.87 (0.44–8.01)	0.41

B, Recessive model.				
	CHD cases vs. non-CHD controls		CHD cases vs. healthy controls	
	OR (95% CI)	P-value	OR (95% CI)	P-value
rs7528419 (GG vs. AG+AA)	-	0.32	-	0.29
rs7756935 (CC vs. CA+AA)	1.21 (0.23–6.36)	1.00	1.03 (0.20–5.41)	1.00
rs1805017 (AA vs. GA+GG)	0.40 (0.13–1.22)	0.13	1.25 (0.25–6.31)	1.00
rs964184 (GG vs. GC+CC)	3.23 (0.72–14.61)	0.16	0.88 (0.32–2.40)	1.00
rs247616 (TT vs. TC+CC)	1.97 (0.41–9.45)	0.51	1.67 (0.35–8.04)	0.73
rs6511720 (TT vs. GT+GG)	-	1.00	-	1.00

CHD, coronary heart disease; OR, odds ratio; CI, confidence interval.

**Table VII.** Logistic regression analysis between SNPs and the severity of CHD.

	Non-CHD controls	No. of arteries			rs7528419	rs7756935	rs1805017	rs964184	rs10846744	rs247616	rs6511720
		1	2	≥3							
Male	**101**	**76**	**50**	**83**	*0.52*	*0.76*	*0.80*	*0.10*	*0.33*	*0.52*	*0.52*
Female	**97**	**29**	**16**	**35**	*0.79*	*0.83*	*0.04*	*0.17*	*0.46*	*0.73*	*0.91*
Total	**198**	**105**	**66**	**118**	*0.68*	*0.84*	*0.38*	*0.05*	*0.32*	*0.46*	*0.77*

Bold results represents the number of patients under corresponding conditions, italic results represent P-values. SNPs, single nucleotide polymorphisms; CHD, coronary heart disease.

## Abbreviations:

SNP: single nucleotide polymorphism;
CHD: coronary heart disease;
HWE: Hardy-Weinberg equilibrium;
GWAS: genome-wide association study;
Lp-PLA_2_: lipoprotein-associated phospholipase A_2_;
OR: odds ratio;
CI: confidence interval
